# NcRNAs: A synergistically antiapoptosis therapeutic tool in Alzheimer's disease

**DOI:** 10.1111/cns.14476

**Published:** 2023-09-22

**Authors:** Liangxian Li, Mingyue Jin, Jie Tan, Bo Xiao

**Affiliations:** ^1^ Laboratory of Respiratory Disease Affiliated Hospital of Guilin Medical University Guilin China; ^2^ Guangxi Key Laboratory of Brain and Cognitive Neuroscience Guilin Medical University Guilin China; ^3^ Key Laboratory of Respiratory Diseases Education Department of Guangxi Zhuang Autonomous Region Guilin China

**Keywords:** AD, cell apoptosis, NcRNAs, regulatory network

## Abstract

**Aims:**

The aim of this review is to systematically summarize and analyze the noncoding RNAs (ncRNAs), especially microRNAs (miRNAs), long noncoding RNAs (lncRNAs), and circular RNAs (circRNAs), in the cell apoptosis among Alzheimer's disease (AD) in recent years to demonstrate their value in the diagnosis and treatment of AD.

**Methods:**

We systematically summarized in vitro and in vivo studies focusing on the ncRNAs in the regulation of cell apoptosis among AD in PubMed, ScienceDirect, and Google Scholar.

**Results:**

We discover three patterns of ncRNAs (including ‘miRNA‐mRNA’, ‘lncRNA‐miRNA‐mRNA’, and ‘circRNA‐miRNA‐mRNA’) form the ncRNA‐based regulatory networks in regulating cell apoptosis in AD.

**Conclusions:**

This review provides a future diagnosis and treatment strategy for AD patients based on ncRNAs.

## Graphical abstract

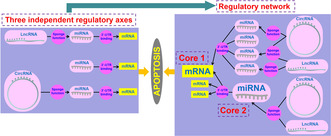

## 1 INTRODUCTION

Alzheimer's disease (AD) is the main cause of dementia, accounting for 60%–80%, and is quickly becoming one of the most expensive, lethal, and burdening diseases of this century.^1^, ^2^ The definition of AD was shifted from a clinical perspective to a biological construct identified by biomarker evidence of both β‐amyloid (Aβ), and pathologic Tau was present in 2018.^3^ The new definition has revolutionized our understanding of AD in clinical diagnosis, helping us take action on AD more quickly. But, at present, the therapeutic strategies mainly focusing on antiamyloid and anti‐Tau have not achieved ideal results.^2^ Therefore, the pathogenesis of AD needs more exploration and the therapeutic strategies of AD need innovation.

Cell apoptosis is defined as a kind of programmed and controlled cell death that accounts for the majority form of cellular death in multiple pathophysiological processes.^4^ Although cell apoptosis is responsible for all death among neurons in AD has not been verified,^5^ it is calculated that the frequency of cell apoptosis is compatible with the rate of progression of neuronal degeneration.^6^ A series of studies confirmed that cells execute apoptosis in postmortem brains from advanced AD patients (Braak stages IV–V) by TUNEL assay.^6^, ^7^, ^8^, ^9^ Therefore, cell apoptosis is an important cause in losing nerve cells among AD brains. Clarifying the mechanism of cell apoptosis is always an important work in diagnosing and treating AD, which cannot be ignored.

Numerous original articles reported that noncoding RNAs (ncRNAs), especially microRNAs (miRNAs), long noncoding RNAs (lncRNAs), and circular RNAs (circRNAs), participate in regulating cell apoptosis in AD in the past decade. NcRNAs have become an important research direction to explore cell apoptosis in AD. This review highlights the role of miRNAs, lncRNAs, and circRNAs in regulating cell apoptosis among AD. Three classic patterns, including ‘miRNA‐mRNA’, ‘lncRNA‐miRNA‐mRNA’, and ‘circRNA‐miRNA‐mRNA’, are discovered. Furthermore, the potential ncRNA‐based networks in regulating cell apoptosis among AD are built. Subsequently, this review proposes an ncRNA‐based synergistically antiapoptosis strategy in investigating the diagnosis and treatment of AD patients.

## 2 CELL APOPTOSIS

**FIGURE 1:**
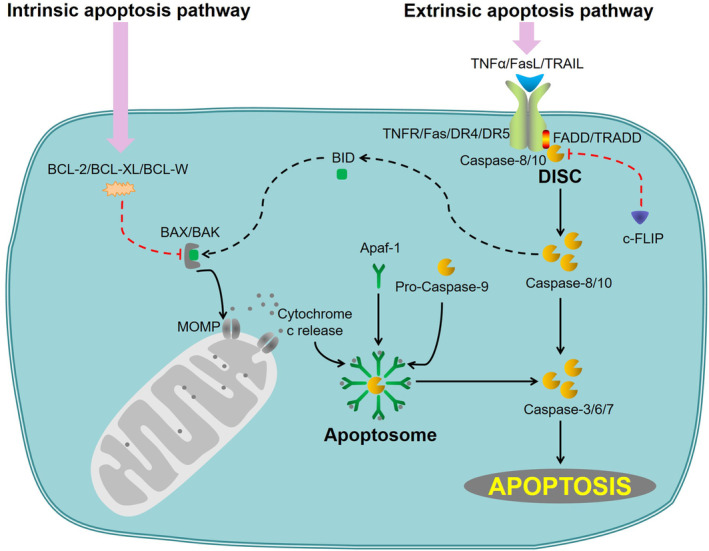
Apoptosis is triggered by the intrinsic and extrinsic pathways. The intrinsic pathway is activated by BAK and BAX. BAK/BAX oligomers form pores, leading to the release of Cytochrome c into the cytosol. Activation of BAK/BAX is promoted by BID or inhibited by BCL‐2/BCL‐XL/BCL‐W. Cytochrome c binds to Apaf‐1, which recruits Pro‐Caspase‐9, forming the apoptosome. In the apoptosome, Pro‐Caspase‐9 is activated by autoproteolytic cleavage, initiating the caspase‐processing cascade. The extrinsic pathway is activated by engagement of membrane receptors. These proteins induce DISC involving TRADD, FADD, Caspase‐8, and Caspase‐10. Initiator Caspase‐8/10 activation is negatively regulated by c‐FLIP. Once active, executioner caspases (Caspase‐3/6/7) bring about apoptosis.

Cell apoptosis was first described as chromatin condensation, nuclear membrane breakdown, cell shrinkage, and formation of small vesicular bodies near the cell surface (apoptotic bodies) in 1972.^10^ It is triggered by two principal pathways referred to as the intrinsic (or mitochondrial) pathway and extrinsic (or death receptor) pathway (Figure 1).

### 2.1 The intrinsic pathway

The intrinsic pathway involves the regulation of mitochondrial outer membrane permeabilization (MOMP) in cell apoptosis by the BCl‐2 family proteins,^11^ which results in the release of Cytochrome c from the mitochondria into the cytosol.^12^ MOMP and release of Cytochrome c are critical requirements to trigger the formation of apoptosomes, which are considered the point of no return in apoptotic cell death.^13^ The release of Cytochrome c is promoted by the proapoptotic BCL‐2 family members, such as BAX, BAK, BIM, and BID.^14^, ^15^, ^16^ Oligomerized BAX and BAK form macropores in the mitochondria membrane, causing MOMP.^14^ BAX and BAK are considered necessary for the execution of cell apoptosis via the intrinsic pathway.^14^ The release of Cytochrome c is inhibited by the antiapoptotic BCL‐2 family members, such as BCL‐2, BCL‐XL, and BCL‐W.^11^, ^17^ These antiapoptotic proteins contain two BCL‐2 homology (BH3) domains, which constitute a binding trench that sequesters BAX and BAK.^18^ The affinity and expression level of BCL‐2 family members ultimately determine the sensitivity or resistance to cell apoptosis.^18^ Cytochrome c, Apaf‐1, and Pro‐Caspase‐9 form the apoptosome in cytosol.^19^, ^20^, ^21^ The mature apoptosome cleaves and activates Pro‐Caspase‐9, initiating the caspase‐processing cascade (activating Pro‐Caspase‐3 and Pro‐Caspase‐7).^22^, ^23^

### 2.2 The extrinsic pathway

The extrinsic pathway is initiated by cell surface death receptors, such as TNFR1/2, Fas, and TRAIL receptors (DR4 and DR5).^24^, ^25^, ^26^ Binding ligands (TNFα, FasL, and TRAIL, respectively) and receptors (TNFR1/2, Fas, DR4, and DR5) cause the accumulation of death receptors on the cell surface.^24^, ^26^, ^27^ Subsequently, the adapter proteins (TRADD and FADD) and the initiator proteins (Caspase‐8 and Caspase‐10) form the death‐inducing signaling complex (DISC).^28^, ^29^, ^30^ The activating of Caspase‐8 and Caspase‐10 is negatively regulated by c‐FLIP in DISC.^31^, ^32^ The activated‐Caspase‐8 and activated‐Caspase‐10 result in the activation of executioners (Caspase‐3, Caspase‐6, and Caspase‐7) either directly through proteolytic cleavage or indirectly by activating the BCL‐2 family member BID.^33^, ^34^ BID is the link between the extrinsic pathway and the intrinsic pathway, which promotes MOMP.^35^

## 3 NCRNAS REGULATE Aβ‐INDUCED CELL APOPTOSIS IN VITRO

SH‐SY5Y cell line, PC12 cell line, SK‐N‐SH cell line, and primary neurons are often used in in vitro studies in AD. The establishment of in vitro models among AD highly depends on the toxicity of Aβ, such as Aβ_42_, Aβ_25‐35_, and Aβ_40_. Many studies have shown that ncRNAs are deeply involved in the regulation of cell apoptosis among AD in recent years, especially in in vitro studies. Therefore, it is necessary to summarize and analyze ncRNAs in regulating cell apoptosis in AD cellular models.

### 3.1 MiRNA‐mRNA

MiR‐124‐3p was significantly downregulated in Aβ_25‐35_‐treated SK‐N‐SH and SK‐N‐BE cells, and BID was upregulated.^36^ Dual‐luciferase reporter assay and RNA pull‐down assay showed that miR‐124‐3p directly targets the 3′‐UTR (untranslated region) of BID.^36^ The miR‐124‐3p‐BID axis reduced cell apoptosis in Aβ_25‐35_‐treated SK‐N‐SH and SK‐N‐BE cells.^36^ Moreover, Aβ_25‐35_ downregulated miR‐137 and upregulated TNFAIP1 in primary mouse cortical neurons and N2a cells.^37^ MiR‐137 directly targeted the 3′‐UTR of TNFAIP1, and miR‐124‐TNFAIP1 axis played an essential role in the inhibition of cell apoptosis induced by Aβ_25‐35_ in primary mouse cortical neurons and N2a cells.^37^ In addition, overexpression of miR‐151‐3p significantly enhanced the antiapoptotic effects in Aβ_42_‐treated SH‐SY5Y and PC12 cells via directly binding to the 3′‐UTR of DAPK‐1 and TP53.^38^ As an inhibitor, miR‐9‐5p was downregulated in Aβ_25‐35_‐treated HT22 cells.^39^ GSK‐3β was a functional target of miR‐9‐5p.^39^ MiR‐9‐5p overexpression inhibited Aβ_25‐35_‐induced cell apoptosis by negatively regulating GSK‐3β expression in HT22 cells.^39^ Similarly, miR‐16‐5p was downregulated in Aβ_42_‐induced SH‐SY5Y and PC12 cells, and primary rat hippocampal neurons.^40^, ^41^ APP and BACE1 were confirmed as the direct targets of miR‐16‐5p.^40^, ^41^ MiR‐16‐5p overexpression repressed Aβ_42_‐induced cell apoptosis by inhibiting the expression of APP and BACE1.^40^, ^41^ Conversely, the expression level of miR‐146a increased in Aβ_42_‐stimulated SH‐SY5Y cells and primary rat cortical neurons.^42^, ^43^ MiR‐146a inhibition suppressed cell apoptosis.^42^, ^43^ Lrp2 was a direct target of miR‐146a.^42^ Overexpression of miRNA‐146a in SH‐SY5Y cells significantly decreased Lrp2 expression, resulting in an induction of proapoptotic Caspase‐3, thereby increasing cell apoptosis.^42^ Table S1 summarizes the miRNAs in regulating cell apoptosis in multiple nerve cells mainly treated with Aβ.^36^, ^37^, ^38^, ^39^, ^40^, ^41^, ^42^, ^43^, ^44^, ^45^, ^46^, ^47^, ^48^, ^49^, ^50^, ^51^, ^52^, ^53^, ^54^, ^55^, ^56^, ^57^, ^58^, ^59^, ^60^ ‘MiRNA‐mRNA’ axis is confirmed as a classic pattern in regulating cell apoptosis in multiple AD cellular models.

### 3.2 LncRNA‐miRNA‐mRNA

LncRNA SNHG1 was upregulated in Aβ_25‐35_‐induced SK‐N‐SH, CHP212, and SH‐SY5Y cells, and human primary neurons.^61^, ^62^ Dual‐luciferase reporter and RNA immunoprecipitation (RIP) assays verified the direct interaction between miR‐361‐3p and SNHG1 or ZNF217.^61^ SNHG1‐miR‐361‐3p‐ZNF217 axis promoted cell apoptosis in Aβ_25‐35_‐induced SK‐N‐SH and CHP212 cells.^61^ Similarly, another SNHG1‐miR‐137‐KREMEN1 axis was confirmed as a facilitator of cell apoptosis in SH‐SY5Y cells and human primary neurons.^62^ In addition, Gu et al. found that lncRNA RPPH1 is upregulated in Aβ_25‐35_‐induced SH‐SY5Y cells.^63^ Dual‐luciferase reporter assay identified the direct interactions between RPPH1 and miR‐326, and miR‐326 and PKM2.^63^ Mechanistically, RPPH1‐miR‐326‐PKM2 axis attenuated cell apoptosis induced by Aβ_25‐35_.^63^ These studies show that ‘lncRNA‐miRNA‐mRNA’ axis is a classic pattern in regulating cell apoptosis in the AD cellular model.

LncRNA SOX21‐AS1 was upregulated in Aβ‐induced (Aβ_25‐35_‐induced and Aβ_42_‐induced) IMR‐32, SH‐SY5Y, and SK‐N‐SH cells.^64^, ^65^ SOX21‐AS1 acted as a sponge for miR‐107 and miR‐137.^64^, ^65^ SOX21‐AS1‐miR‐107 axis and SOX21‐AS1‐miR‐137 axis promoted cell apoptosis in IMR‐32, SH‐SY5Y, and SK‐N‐SH cells induced by Aβ.^64^, ^65^ In addition, lncRNA ANRIL was upregulated in Aβ_42_‐induced PC12 cells.^66^ Similarly, the ANRIL‐miR‐125a axis was confirmed as a promoter of cell apoptosis in Aβ_42_‐induced PC12 cells.^66^ Moreover, lncRNA NEAT1 was overexpressed in Aβ_42_‐induced SH‐SY5Y and SK‐N‐SH cells.^67^ The direct interaction between NEAT1 and miR‐107 was verified by dual‐luciferase reporter and RIP assays.^67^ NEAT1‐miR‐107 axis accelerated the promotion of cell apoptosis in Aβ_42_‐induced SH‐SY5Y and SK‐N‐SH cells.^67^ As a promoter, the enrichment of lncRNA BACE1‐AS was positively regulated by Aβ_25‐35_ in HPN and SK‐N‐SH cells.^68^ The binding site between miR‐132‐3p and BACE1‐AS was confirmed by dual‐luciferase reporter assay.^68^ BACE1‐AS‐miR‐132‐3p axis promoted cell apoptosis in Aβ_25‐35_‐induced HPN and SK‐N‐SH cells.^68^ The above studies require further research to identify the target mRNAs of ‘lncRNA‐miRNA’ axes. Subsequently, the complete ‘lncRNA‐miRNA‐mRNA’ axes will be verified in regulating cell apoptosis in the AD cellular models.

The direct interaction between lncRNA RP11‐543N12.1 and miR‐324‐3p was verified by dual‐luciferase reporter assay.^69^ However, RP11‐543N12.1 and miR‐324‐3p were both upregulated in Aβ_25‐35_‐induced SH‐SY5Y cells.^69^ RP11‐543N12.1 and miR‐324‐3p both contributed to promotion of cell apoptosis in Aβ_25‐35_‐induced SH‐SY5Y cells.^69^ It means RP11‐543N12.1 may act as a stabilizer of miR‐324‐3p and upregulate its expression. Furthermore, this is not a classic pattern as previously described in regulating cell apoptosis in the AD cellular model.

Additionally, lncRNA MALAT1,^70^ lncRNA BDNF‐AS,^71^ lncRNA BC200,^72^, ^73^ and lncRNA 17A^74^ were closely related to cell apoptosis in in vitro studies in AD. However, whether they regulate cell apoptosis through ‘lncRNA‐miRNA‐mRNA’ axis has not been explored. We need to solve the mystery left behind.

### 3.3 CircRNA‐miRNA‐mRNA

Circ‐AXL was upregulated in Aβ_42_‐treated SK‐N‐SH and SH‐SY5Y cells.^75^, ^76^ RIP and dual‐luciferase reporter assays presented that circ‐AXL directly bind miR‐1306‐5p, and miR‐1306‐5p directly targets the 3′‐UTR of PDE4A.^75^ Circ‐AXL‐miR‐1306‐5p‐PDE4A axis promoted cell apoptosis in Aβ_42_‐treated SK‐N‐SH cells.^75^ Similarly, another circ‐AXL‐miR‐328‐BACE1 axis was also verified to promote cell apoptosis in Aβ_42_‐treated SK‐N‐SH and SH‐SY5Y cells.^76^ The above studies indicate that ‘circRNA‐miRNA‐mRNA’ axis is a classic pattern in regulating cell apoptosis in the AD cellular model.

Aβ_42_ triggered a significant downregulation in circHDAC9 and a striking upregulation in miR‐142‐5p in human neuronal cells.^77^ The direct interaction between circHDAC9 and miR‐142‐5p was confirmed by dual‐luciferase reporter, RIP, and RNA pull‐down assays.^77^ CircHDAC9‐miR‐142‐5p axis inhibited cell apoptosis in Aβ_42_‐treated human neuronal cells.^77^ The expression level of circ_0000950 was not changed in PC12 cells and rat primary cerebral cortex neurons.^78^ However, overexpression of circ_0000950 aggravated Aβ_42_‐induced cell apoptosis in PC12 cells and rat primary cerebral cortex neurons.^78^ Compensation experiment and dual‐luciferase reporter assay further validated the sponging effect of circ_0000950 on miR‐103 as well as the mechanism of circ_0000950‐miR‐103 axis on promoting cell apoptosis.^78^ The above studies require further research to identify the direct target mRNAs of ‘lncRNA‐miRNA’ axes. Then, the complete ‘lncRNA‐miRNA‐mRNA’ axes will be verified in regulating cell apoptosis in the AD cellular model.

## 4 ABNORMALLY EXPRESSED NCRNAS IN AD ANIMAL MODELS IN REGULATING CELL APOPTOSIS IN AD

Transgene (such as APP/PS1, 5xFAD, and SAMP8) and microinjection (such as Aβ_42_, Aβ_25‐35_, and Aβ_40_) are often used to construct AD animal models.^79^ Many studies have shown that numerous abnormally expressed ncRNAs in AD animal models are deeply involved in regulating cell apoptosis in AD. Therefore, it is necessary to summarize and analyze it.

### 4.1 MiRNA‐mRNA

MiR‐429 was upregulated, and SOX2 and BCL‐2 were downregulated in the AD mouse model (APPswe/PSΔE9 double transgenic mice).^80^ Dual‐luciferase reporter assay indicated that SOX2 and BCL‐2 are direct targets of miR‐429.^80^ MiR‐429‐SOX2 axis and miR‐429‐BCL‐2 axis promoted cell apoptosis in Aβ_25‐35_‐induced primary mouse cortical neurons.^80^ Similarly, the expression level of miR‐142‐5p was increased in the AD animal models (APPswe/PS1dE9 double transgenic mice and injection of Aβ_42_ oligomer once into the lateral ventricle of rats).^81^, ^82^ BAI3 was a direct target of miR‐142‐5p,^81^ and PTPN1 was also a direct target of miR‐142‐5p.^82^ MiR‐142‐5p‐PTPN1 axis and MiR‐142‐5p‐BAI3 axis promoted cell apoptosis in AD rats and Aβ_42_‐induced HT‐22 cells, respectively.^81^, ^82^ In contrast, miR‐107 was downregulated in APP/PS1 double transgenic mice^83^ and 6‐hydroxydopamine (6‐OHDA)‐insulted mice.^84^ PDCD10 was a direct target of miR‐107.^84^ MiR‐107‐PDCD10 axis inhibited cell apoptosis in 6‐OHDA‐treated SH‐SY5Y and PC12 cells.^84^ In addition, miR‐132‐3p presented a low expression state in the AD rat models established by intracerebroventricular administration of Aβ_25‐35_ ^85^ and tail vein injection of homocysteine.^86^ MAPK1 was a direct target of miR‐132‐3p,^85^ and HNRNPU was also a direct target of miR‐132‐3p.^86^ MiR‐132‐3p‐MAPK1 axis and miR‐132‐3p‐HNRNPU axis inhibited cell apoptosis in the AD rat models.^85^, ^86^ Table S2 summarizes all the cell apoptosis‐related and abnormally expressed miRNAs in multiple AD animal models.^80^, ^81^, ^83^, ^84^, ^85^, ^86^, ^87^, ^88^, ^89^, ^90^, ^91^, ^92^, ^93^, ^94^, ^95^, ^96^, ^97^, ^98^, ^99^, ^100^, ^101^, ^102^, ^103^, ^104^, ^105^, ^106^, ^107^, ^108^, ^109^, ^110^, ^111^, ^112^, ^113^, ^114^, ^115^, ^116^, ^117^, ^118^, ^119^, ^120^ These miRNAs can form ‘miRNA‐mRNA’ axis in regulating cell apoptosis in AD.

Interestingly, the relationship between miR‐34a and cell apoptosis in AD required special attention. Wang et al. and Li et al. showed that the expression level of miR‐34a is increased in APPswe/PSΔE9 mice and PS‐2 mutant (N141I) transgenic mice.^87^, ^89^ BCL‐2 and SIRT1 were both the targets of miR‐34a.^87^, ^89^ MiR‐34a promoted cell apoptosis by negatively regulating the expression of BCL‐2 and SIRT1.^87^, ^89^ Conversely, Modi et al. showed that miR‐34a expression increases significantly until 6 months, followed by a decrease, which was significantly lower than in the WT (wild‐type) animals aged more than 12 months in APP/PS1 transgenic mice.^88^ Cyclin D1 was a direct target of miR‐34a.^88^ MiR‐34a inhibited cell apoptosis by negatively regulating the expression of Cyclin D1.^88^ We noticed that Wang et al. used 3‐month‐old and 6‐month‐old transgenic mice in their study,^87^ and Li et al. used 9‐month‐old transgenic mice in their study.^89^ And Modi et al. found that miR‐34a expression increases significantly until 6‐month‐old, followed by a decrease more than 12‐month‐old, in the transgenic mice.^88^ Therefore, whether miR‐34a promotes or inhibits cell apoptosis in AD may be related to the age of the transgenic mice.

### 4.2 LncRNA‐miRNA‐mRNA

LncRNA NEAT1 was upregulated, while miR‐27a‐3p and miR‐124 were downregulated in AD mouse model (microinjection of streptozocin) and AD rat model (microinjection of Aβ_40_).^121^, ^122^ Dual‐luciferase reporter assay showed that NEAT1 can directly target miR‐124, and miR‐124 can directly target 3′‐UTR of BACE1.^121^ Dual‐luciferase reporter assay and RNA pull‐down experiment showed that NEAT1 can also directly target miR‐27a‐3p.^122^ NEAT1‐miR‐124‐BACE1 axis and NEAT1‐miR‐27a‐3p axis promoted cell apoptosis in Aβ_42_‐indcued PC12 cells and Aβ_40_‐indcued SH‐SY5Y cells, respectively.^121^, ^122^ Similarly, lncRNA H19 and HMGB1 expressions were elevated while miR‐129 expression was reduced in brain tissues of AD mice (microinjection of Aβ_25‐35_).^123^ MiR‐129 was a direct target of H19, and HMGB1 was a direct target of miR‐129.^123^ Silencing H19‐miR‐129‐HMGB1 axis repressed cell apoptosis in Aβ_25‐35_‐indcued PC12 cells, which might be beneficial for AD treatment.^123^ Moreover, lncRNA RPPH1 and miR‐122 were both dramatically elevated in cortical samples of AD mouse model (APPswe/PS1∆E9 double transgenic mice).^124^ Dual‐luciferase reporter assay confirmed the binding of miR‐122 with predictive binding sites in RPPH1 and Wnt1.^124^ RPPH1‐miR‐122‐Wnt1 axis inhibited cell apoptosis via activating Wnt/β‐catenin signaling in Aβ_25‐35_‐induced SK‐N‐SH cells.^124^ Additionally, lncRNA MALAT1 and CNR1 were poorly expressed while miR‐30b was highly expressed in AD rats.^125^ MALAT1 served as a sponge for miR‐30b, and miR‐30b directly targeted 3′‐UTR of CNR1.^125^ MALAT1‐miR‐30b‐CNR1 axis inhibited cell apoptosis in PC12 and C6 cells.^125^ These studies show that the abnormally expressed miRNAs in AD animal models can form ‘lncRNA‐miRNA‐mRNA’ axis in regulating cell apoptosis in AD.

The expression of lncRNA EBF3‐AS was upregulated in hippocampus of APP/PS1 mice.^126^ EBF3‐AS promoted cell apoptosis in Aβ_25‐35_‐induced SH‐SY5Y cells and might be a new therapeutic target for treatment of AD.^126^ In addition, downregulated lncRNA MEG3 was detected in the hippocampus tissues of AD rats (microinjection of Aβ_25‐35_).^127^ Upregulation of MEG3 inhibited cell apoptosis in hippocampal neurons in AD rats.^127^ EBF3‐AS and MEG3 are closely related to cell apoptosis in AD. However, whether they regulate cell apoptosis through ‘lncRNA‐miRNA‐mRNA’ axis has not been explored. We need to solve the mystery left behind.

Interestingly, lncRNA n336694 and miR‐106b were both upregulated in APP/PS1 double transgenic mice.^128^ Dual‐luciferase reporter assay showed that n336694 directly targets the 3′‐UTR of miR‐106b.^128^ Overexpression of n336694 obviously increased the expression of miR‐106b.^128^ Subsequently, transfecting miR‐106b mimics promoted cell apoptosis in SH‐SY5Y cells.^128^ Moreover, bioinformatics analysis revealed that lncRNA SOX21‐AS1 was upregulated in AD.^129^ SOX21‐AS1 directly targeted the 3′‐UTR of FZD3/5.^129^ Silencing of SOX21‐AS1 could act to alleviate neuronal apoptosis in AD mice through the upregulation of FZD3/5 and subsequent activation of the Wnt signaling pathway.^129^ Similarly, the expression of lncRNA XIST was increased in AD mice.^130^ RIP validated the combination of XIST and EZH2.^130^ XIST promoted cell apoptosis by negatively regulating EZH2 in AD.^130^ Conversely, the expression of lncRNA WT1‐AS was decreased in the brain tissues of Aβ_25‐35_‐induced mice.^131^ WT1‐AS overexpression inhibited WT1 expression, and WT1 could directly target the promoter region of miR‐375 to promote its expression.^131^ MiR‐375 could bind the 3′‐UTR of SIX4, and miR‐375‐SIX4 axis promoted cell apoptosis in SH‐SY5Y cells.^131^ These studies show that the abnormally expressed lncRNAs in the AD animal models can regulate cell apoptosis in AD by these nonclassic patterns. These nonclassic patterns are independent on the ‘lncRNA‐miRNA‐mRNA’ axis described previously.

## 5 ABNORMALLY EXPRESSED NCRNAS IN AD PATIENTS IN REGULATING CELL APOPTOSIS IN AD

No matter what role ncRNAs play on cell apoptosis in cellular AD models and AD animal models, we should first verify whether the related ncRNAs are abnormally expressed in AD patients. In other words, if the expression of these ncRNAs does not change in AD patients, it would be a devastating blow to relevant research. Many studies have shown that numerous ncRNAs, which are abnormally expressed in AD patients, are deeply involved in regulating cell apoptosis in AD in recent years. Therefore, it is necessary to summarize and analyze it.

### 5.1 miRNA‐mRNA

MiR‐212 downregulated in human plasma and brains with AD.^132^, ^133^ PTEN and FOXO3a were direct targets of miR‐212,^132^ and PDCD4 was also a direct target of miR‐212.^133^ MiR‐212‐PTEN/FOXO3 axes^132^ and miR‐212‐PDCD4 axis^133^ reduced neuronal apoptosis and contributed to AD neuroprotection. Additionally, a decreased expression of miR‐129 was detected in serum of AD patients.^134^ MiR‐129 directly targeted YAP1 and disrupted its interaction with JAG1, leading to a decline in hippocampal neuron apoptosis and attenuated cognitive impairment in Aβ_42_‐injected mice.^134^ In other words, miR‐129‐YAP1 axis might be a new therapeutic target for AD treatment.^134^ Moreover, the expression of miR‐539‐5p was significantly downregulated in human cerebrospinal fluid (CSF) with AD, upregulation of miR‐539‐5p inhibited cell apoptosis in APP/PS1 transgenic mice.^135^ Dual‐luciferase reporter assay showed that APP, CAV1, and GSK‐3β are direct targets of miR‐539‐5p.^135^ MiR‐539‐5p was negatively correlated with the expression of APP, CAV1, and GSK‐3β, and miR‐539‐5p‐APP/CAV1/GSK‐3β axes might be a novel pathologic mechanism in regulating cell apoptosis in APP/PS1 transgenic mice.^135^ Conversely, miR‐26b significantly elevated in the defined pathological areas of human postmortem brains, starting from early stages of AD (Braak III).^136^ Rb1 was a direct target of miR‐26b.^136^ MiR‐26b‐Rb1 axis promoted cell apoptosis and inhibited cell viability in human primary cortical neurons.^136^ Similarly, miR‐425‐5p was upregulated in human postmortem brain with AD.^137^ HSPB8 was directly targeted by miR‐425‐5p.^137^ MiR‐425‐5p‐HSPB8 axis induced cell apoptosis and promoted Tau phosphorylation in AD, and might act as a new therapeutic target for AD treatment.^137^ In addition, miR‐485‐3p was overexpressed in the brain tissues, CSF, and plasma of patients with AD.^138^ CD36 was a direct target of miR‐485‐3p.^138^ MiR‐485‐3p‐CD36 axis weakened the phagocytosis of Aβ in vitro and in vivo and might promote cell apoptosis in AD.^138^ Table S3 summarizes all the abnormally expressed and cell apoptosis‐related miRNAs in AD patients.^132^, ^133^, ^134^, ^135^, ^136^, ^137^, ^138^, ^139^, ^140^, ^141^, ^142^, ^143^, ^144^, ^145^, ^146^, ^147^, ^148^, ^149^, ^150^, ^151^, ^152^, ^153^, ^154^, ^155^, ^156^, ^157^, ^158^, ^159^, ^160^ These miRNAs can form ‘miRNA‐mRNA’ axis in regulating cell apoptosis in AD.

Interestingly, miR‐132 downregulated in human AD brains.^132^ PTEN and FOXO3a were both the direct targets of miR‐132.^132^ MiR‐132‐PTEN/FOXO3 axes reduced neuronal apoptosis and contributed to AD neuroprotection.^132^ However, Liu et al. showed that the expression of miR‐132 is significantly higher in human AD brains.^139^ Dual‐luciferase reporter assay verified that GTDC‐1 is a direct target of miR‐132.^139^ MiR‐132‐GTDC‐1 axis promoted cell apoptosis in human primary cortical neurons.^139^ Similarly, the expression level of miR‐125b‐5p was increased in the CSF samples of AD patients^140^ but decreased in the serum samples of AD patients.^141^ Jin et al. confirmed that miR‐125b‐5p promotes cell apoptosis in Neuro2a APPswe/Δ9 cells.^140^ Li et al. confirmed that BACE1 acts as a direct target of miR‐125b‐5p, and miR‐125b‐5p‐BACE1 axis inhibited cell apoptosis in Aβ_25‐35_‐treated MCN and N2a cells.^141^ These studies showed that the true expression and function of miR‐132 and miR‐125b‐5p need to be further confirmed in AD.

### 5.2 LncRNA‐miRNA‐mRNA

LncRNA ATB was overexpressed in the CSF and serum of AD patients.^161^ ATB negatively regulated the expression of miR‐200, and suppression of ATB alleviated Aβ_25‐35_‐induced cell apoptosis by regulation of miR‐200 in PC12 cells.^161^ ZNF217 was a direct target of miR‐200.^161^ ATB might protect PC12 cells against Aβ_25‐35_‐induced cell apoptosis via regulating miR‐200/ZNF217 axis, and ATB‐miR‐200‐ZNF217 axis may provide a new insight for preventing AD.^161^ Similarly, lncRNA RMRP was upregulated in serum of AD patients.^162^ Dual‐luciferase reporter assay showed that RMRP acts as a sponge of miR‐3142, and miR‐3142 directly targets TRIB3.^162^ RMRP‐miR‐3142‐TRIB3 axis promoted cell apoptosis in Aβ_42_‐inudced SH‐SY5Y cells.^162^ These above lncRNAs abnormally express in AD patients, and the ‘lncRNA‐miRNA‐mRNA’ axis is also a classic pattern in regulating cell apoptosis in AD.

LncRNA RP11‐59J16.2 was upregulated in serum of AD patients.^163^ Interestingly, dual‐luciferase reporter assay verified that RP11‐59J16.2 can directly interact with 3′‐UTR of MCM2 and further downregulated the expression of MCM2.^163^ RP11‐59J16.2 aggravated Aβ_42_‐induced cell apoptosis by negatively regulating the expression of MCM2 in SH‐SY5Y cells.^163^ This indicates that lncRNAs can also directly target mRNA and regulate cell apoptosis in AD. In other words, ‘lncRNA‐mRNA’ axis may be another direction of exploration in regulating cell apoptosis in AD.

### 5.3 CircRNA‐miRNA‐mRNA

Circ‐HUWE1 was increased in serum samples of AD patients.^164^ The predicted binding relationship between miR‐433‐3p and circ‐HUWE1 or FGF7 was validated by dual‐luciferase reporter assay.^164^ Circ‐HUWE1‐miR‐433‐3p‐FGF7 axis could promote cell apoptosis in Aβ_40_‐induced SK‐N‐SH cells.^164^ Similarly, the expression of circ_0003611 was markedly upregulated in the serum of patients with AD relative to healthy controls.^165^ RIP and dual‐luciferase reporter assay identified that circ_0003611 works as a miR‐383‐5p sponge, and miR‐383‐5p directly targets KIF1B.^165^ Circ_0003611‐miR‐383‐5p‐KIF1B axis promoted cell apoptosis in Aβ‐triggered SH‐SY5Y and SK‐N‐SH cells.^165^ Moreover, circ_0002945 was overexpressed in serum of AD patients.^166^ TNFAIP1 was a direct target of miR‐431‐5p and circ_0002945 functioned as a ceRNA to control TNFAIP1 expression via miR‐431‐5p competition.^166^ Inhibition of circ_0002945‐miR‐431‐5p‐TNFAIP1 axis attenuated Aβ_25‐35_‐induced cell apoptosis in human primary neurons and SK‐N‐SH cells.^166^ Additionally, circLPAR1 was highly expressed in the blood of AD patients.^167^ The direct interaction between miR‐212‐3p and ZNF217 or circLPAR1 was verified by dual‐luciferase reporter and RIP assays.^167^ CircLPAR1‐miR‐212‐3p‐ZNF217 axis promoted Aβ_25‐35_‐induced cell apoptosis in CHP‐212 and IMR‐32 cells, suggesting a new insight into the pathogenesis of AD.^167^ Furthermore, circ_0049472 was overexpressed in CSF and serum of AD patients.^168^ MiR‐107 was a direct target of circ_0049472, and KIF1B was a direct target of miR‐107.^168^ Circ_0049472‐miR‐212‐3p‐KIF1B axis promoted cell apoptosis in Aβ‐induced SK‐N‐SH and CHP‐212 cells.^168^ These circRNAs work as promoters of cell apoptosis in AD, suggesting a potential ‘circRNA‐miRNA‐mRNA’ axis in AD pathogenesis.

## 6 THE NCRNA‐BASED ANTIAPOPTOSIS REGULATORY NETWORKS IN AD

**FIGURE 2:**
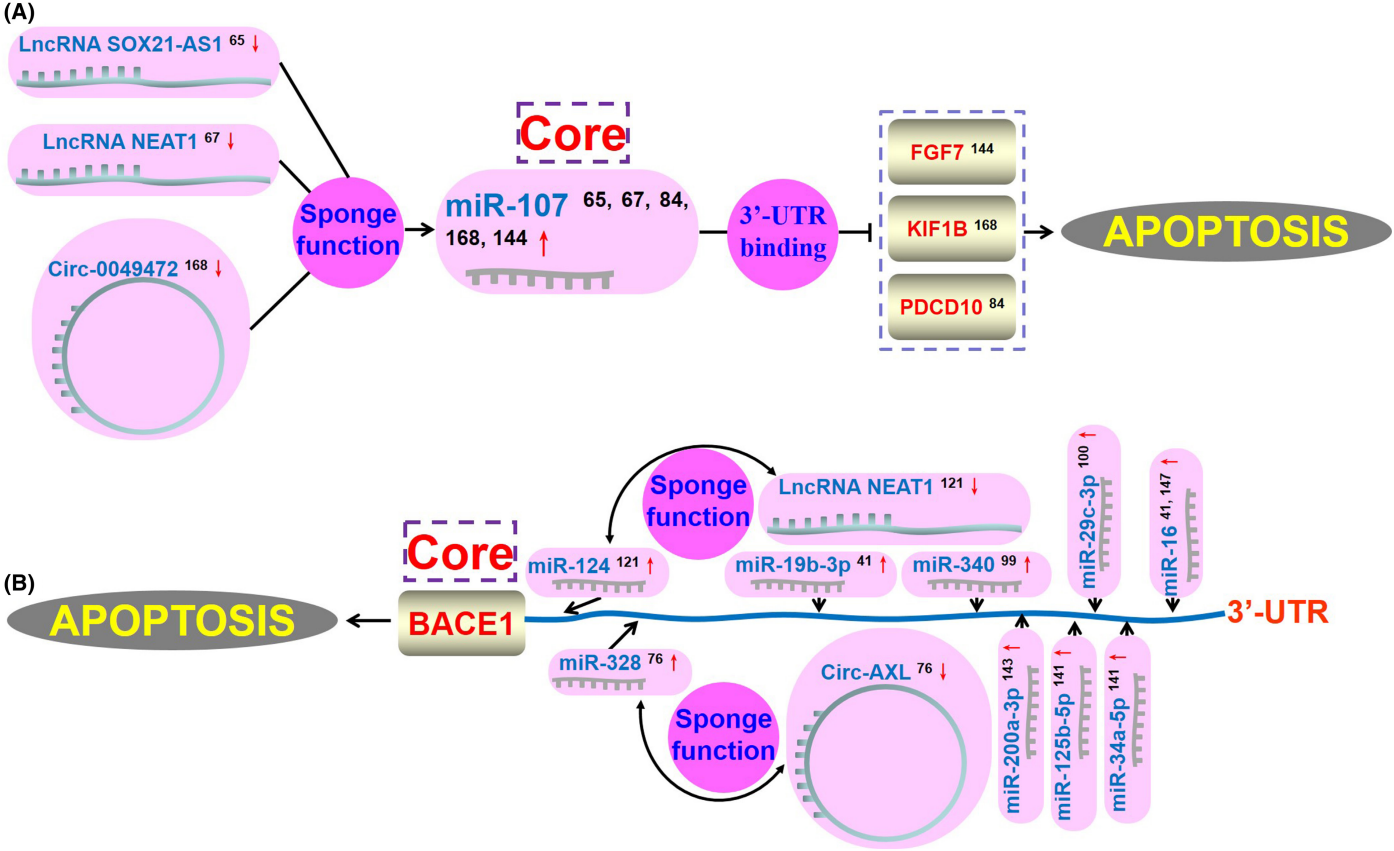
The potential synergistically antiapoptosis ncRNA‐based regulatory networks exist in AD. (A) Based on miR‐107, the network is constructed in regulating cell apoptosis in AD and the simple antiapoptotic strategy is proposed. (B) Based on BACE1, the network is constructed in regulating cell apoptosis in AD and the simple antiapoptotic strategy is proposed. Red arrows (‘
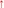
’ and ‘
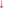
’) present for upregulation and downregulation, respectively.

All the abovementioned studies are related to the abnormally expressed ncRNAs in cellular AD models, AD animal models, and AD patients, and their function in regulating cell apoptosis in AD. Therefore, we can build the potential and broader ncRNA‐based antiapoptosis regulatory networks based on ‘miRNA’ or ‘mRNA’ as the core, respectively. Figure 2A shows an ncRNA‐based antiapoptosis regulatory network constructed with miR‐107 as the core. Downregulation of lncRNA SOX21‐AS1, lncRNA NEAT1, and circ_0049472 and upregulation of miR‐107 should exert synergistic antiapoptosis effects in AD (Figure 2A). Figure 2B shows an ncRNA‐based antiapoptosis regulatory network constructed with BACE1 as the core. Downregulation of lncRNA NEAT1 and circ‐AXL, and upregulation of miR‐124, miR‐328, miR‐200a‐3p, miR‐340, miR‐19b‐3p, miR‐125b‐5p, miR‐34a‐5p, miR‐29c‐3p, and miR‐16 should exert synergistic antiapoptosis effects in AD (Figure 2B).

Tables S4 and S5 summarize other ncRNAs that synergistically inhibit cell apoptosis in AD with miRNAs or mRNAs as the cores. Although these ncRNAs cannot form a broad regulatory network as miR‐107 and BACE1, they also deserve our attention nonetheless.

**FIGURE 3:**
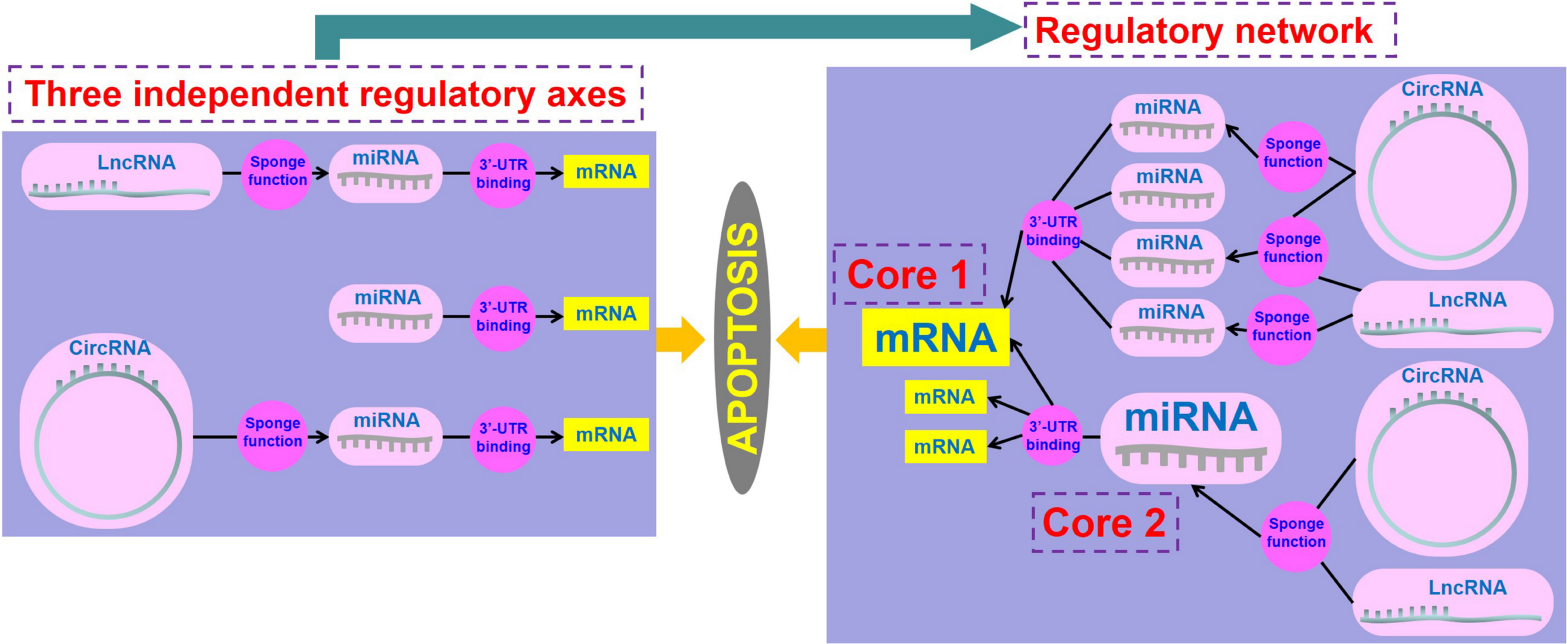
The potential synergistically ncRNA‐based apoptosis regulatory mechanism exists in AD. First, three independent regulatory axes in left, ‘miRNA‐mRNA’, ‘lncRNA‐miRNA‐mRNA’, and ‘circRNA‐miRNA‐mRNA’, regulate cell apoptosis in AD. Then, intertwined ncRNA‐based regulatory network in right regulates cell apoptosis in AD.

More importantly, based on the three core regulatory axes, ‘miRNA‐mRNA’, ‘lncRNA‐miRNA‐mRNA’, and ‘circRNA‐miRNA‐mRNA’, this review constructs a schematic diagram of a broader regulatory network that helps us understand better the mechanism by which ncRNAs involved in cell apoptosis of AD (Figure 3). As the studies on the cell apoptosis regulatory in AD by ncRNAs become more and more abundant, the ncRNA‐based cell apoptosis regulatory network map will become more and more complete, and our ncRNA‐based strategies for the diagnosis and treatment of cell apoptosis in AD will also become more and more perfect.

## 7 DISCUSSION

At present, studies on the roles of ncRNAs in regulating cell apoptosis of AD and their clinical relevance are still in the development stage. Particularly, transcriptomic studies provided us with a large number of abnormally expressed ncRNAs.^169^, ^170^, ^171^, ^172^, ^173^ The identification of whether these ncRNAs participate in regulating cell apoptosis in AD will be our important work. The verification of the functions of the above ncRNAs will greatly expand the breadth of ncRNAs in regulating cell apoptosis in AD.

Many studies demonstrated that numerous cell apoptosis‐related and abnormally expressed ncRNAs in AD also participate in regulating Aβ accumulation, Tau phosphorylation, inflammation, oxidative stress, and so on. For example, downregulation of miR‐98‐5p decreased SNX6‐dependent levels of Aβ_40_, Aβ_42_, BACE1, soluble amyloid precursor protein β (sAPPβ), and membrane‐associated APP β‐carboxyl terminal fragment (βCTF) in SK‐N‐SH cells.^54^ In other words, the cell apoptosis‐related miR‐98‐5p played a critical role in accumulation of Aβ in AD.^54^ Overexpression of miR‐132 induced neuronal apoptosis by increasing BAX and decreasing BCL‐2 and also upregulated phosphorylation of Tau.^136^ Sequence‐specific inhibition of miR‐132 and miR‐212 induced cell apoptosis in cultured primary neurons, whereas their overexpression was neuroprotective against oxidative stress.^132^ Circ_0000950 promoted neuron apoptosis, suppressed neurite outgrowth, and elevated inflammatory cytokine levels through directly sponging miR‐103 in AD.^139^ These studies imply that when we develop the antiapoptosis strategies based on ncRNAs, we may gain additional benefits in against Aβ accumulation, Tau phosphorylation, inflammation, oxidative stress, and so on. Concurrently, we should be aware that we may find a large number of ncRNAs involved in the regulation of cell apoptosis in the studies focusing on ncRNAs in regulating other AD‐related pathological changes. It will help us amplify the ncRNA‐based cell apoptosis regulatory network in AD and establish a more perfect antiapoptosis strategy in AD.

In future, as more ncRNAs in cell apoptosis regulatory of AD are identified, a more complete ncRNA‐based regulatory network will be built. Based on this gradually improved network, we will have a more complete understanding of ncRNAs in cell apoptosis regulatory of AD. This will help us formulate ncRNA‐based antiapoptosis synergistic strategies and improve the role of ncRNAs in the diagnosis and treatment of AD.

In addition, three suggestions were proposed for future research on the role of ncRNAs in cell apoptosis regulatory among AD. First, Jin et al. found that miR‐125b‐5p induced cell apoptosis in Neuro2a APPswe/Δ9 cells,^140^ but Li et al.^141^ showed that miR‐125b‐5p inhibited cell apoptosis in Aβ_25‐35_‐induced primary mouse cortical neurons and Neuro2a cells. In these cases, more studies are needed to confirm the real role of such ncRNAs. Second, future studies can focus on whether ncRNAs that function in other AD pathological changes affect the cell apoptosis in AD. Third, future studies can focus on lncRNAs and circRNAs in regulating cell apoptosis among AD. After all, there are fewer studies on lncRNAs and circRNAs comparing with miRNAs so far.

Moreover, Cai et al.^69^ showed that lncRNA RP11‐543N12.1 and miR‐324‐3p are both upregulated in Aβ_25‐35_‐induced SH‐SY5Y cells, and RP11‐543N12.1 directly targets miR‐324‐3p. Unusually, overexpressed RP11‐543N12.1 upregulated the expression of miR‐324‐3p, and RP11‐543N12.1 and miR‐324‐3p both promoted cell apoptosis in Aβ_25‐35_‐induced SH‐SY5Y cells.^69^ Additionally, Huang et al. found that lncRNA n336694 and miR‐106b are overexpressed in APP/PS1 mice brain tissues, and miR‐106b is a direct target of n336694.^128^ Unconventionally, overexpressed n336694 upregulated the expression of miR‐106b, and n336694 and miR‐106b both promoted cell apoptosis in SH‐SY5Y cells.^128^ The two articles show that lncRNAs may act as a stabilizer of miRNAs and upregulate its expression. Otherwise, bioinformatics analysis revealed that lncRNA SOX21‐AS1 was upregulated in AD.^129^ SOX21‐AS1 directly targeted the 3′‐UTR of FZD3/5.^129^ Silencing of SOX21‐AS1 alleviated neuronal apoptosis in AD mice through the upregulation of FZD3/5.^129^ Similarly, the expression of lncRNA XIST was increased in AD mice.^130^ RIP validated the combination of XIST and EZH2.^130^ XIST promoted cell apoptosis by negatively regulating EZH2 in AD.^130^ The two articles show that ‘lncRNA‐mRNA’ axis can also regulate cell apoptosis in AD. These ncRNA‐based nonclassic patterns in regulating cell apoptosis in AD are an important supplements for our networks described previously. It will help us understand better ncRNAs in regulating cell apoptosis in AD.

## 8 CONCLUSIONS

This review summarizes for the first time that the mechanisms which ncRNAs regulate cell apoptosis in AD. We propose to build the regulatory network based on the three classical regulatory patterns in helping us understand better ncRNAs in regulating cell apoptosis in AD. Subsequently, the network facilitates the construction of ncRNA‐based synergistically diagnostic and therapeutic strategies for cell apoptosis in AD. Finally, we must be aware that such ncRNA‐based network can also be constructed in different diseases. It will improve our understanding of ncRNAs in regulating the occurrence and development of various diseases, and help formulate a more effective and reasonable ncRNA‐based diagnosis and treatment strategies.

## AUTHOR CONTRIBUTIONS

Liangxian Li contributed to conceptualization, methodology, software, data curation, and original draft preparation. Liangxian Li and Mingyue Jin contributed to visualization and investigation. Jie Tan and Bo Xiao contributed to supervision, writing, reviewing, and editing. All authors read and approved the final manuscript document.

## FUNDING INFORMATION

This work was supported by the Open Project Program of Guangxi Key Laboratory of Brain and Cognitive Neuroscience (GKLBCN‐20190103, GKLBCN‐20190105‐04, and GKLBCN‐202106‐02) and the Young and Middle‐Aged Teachers' Basic Research Ability Improvement Project of Universities in Guangxi (2023KY0522 and 2023KY0541).

## CONFLICT OF INTEREST STATEMENT

The authors have no conflicts of interest to declare.

## Supporting information


Table S1

Table S2

Table S3

Table S4

Table S5

## Data Availability

Data sharing does not apply to this article, and this research does not involve the analysis and innovation of new data.
