# Supplementary material for: NcRNAs: A synergistically antiapoptosis therapeutic tool in Alzheimer's disease
Source: CNS Neurosci Ther. 2023 Sep 22;30(4):e14476. doi: 10.1111/cns.14476 (PMC11017435; doi:10.1111/cns.14476)
Supplement: Supplementary file 1 — Table S1 [file CNS-30-e14476-s001.doc]

**Supplementary Table 1** Differentially expressed miRNAs in different cellular AD models and their apoptosis regulation.

| Cellular AD model | | MiRNA | Expression | Target | Target validation methods | Apoptosis | References |
| --- | --- | --- | --- | --- | --- | --- | --- |
| Cell type | Stimulation |
| SK‑N‑SH cell line and SK‑N‑BE cell line | Aβ_25-35_ | MiR-124-3p | Down-regulated | BID | Dual-luciferase reporter and RNA pull-down assays | ↓ | 36 |
| SH-SY5Y cell line | Aβ_42_ | MiR-146a | Up-regulated | Lrp2 | Dual-luciferase reporter assay | ↑ | 42 |
| HT22 cell line | Aβ_25-35_ | MiR-9-5p | Down-regulated | GSK-3β | Dual-luciferase reporter assay | ↓ | 39 |
| Primary mouse cortical neurons and N2a cell line | Aβ_25-35_ | MiR-137 | Down-regulated | TNFAIP1 | Dual-luciferase reporter assay | ↓ | 37 |
| PC12 cell line and primary rat hippocampal neurons | Aβ_42_ | MiR-16-5p | Down-regulated | APP | Dual-luciferase reporter assay | ↓ | 40 |
| SH-SY5Y cell line | Aβ_42_ | MiR-16-5p | Down-regulated | BACE1 | RIP and dual-luciferase reporter assays | ↓ | 41 |
| SH-SY5Y cell line | Aβ_25-35_ | MiR-377 | Down-regulated | CDH13 | Dual-luciferase reporter assay | ↓ | 44 |
| Primary rat hippocampal neurons | Aβ_42_ | MiR-194 | Up-regulated | Nrn1 | Dual-luciferase reporter assay | ↑ | 45 |
| SK-N-SH cell line and CHP 212 cell line | Aβ_42_ | MiR-29c-3p | Down-regulated | TNFAIP1 | RIP and dual-luciferase reporter assays | ↓ | 46 |
| SH-SY5Y cell line | Aβ_42_ | MiRNA-138 | Up-regulated | DEK | Dual-luciferase reporter assay | ↑ | 47 |
| BV2 and N2a cell line | Aβ_25-35_ | MiR-188 | Down-regulated | NOS1 | RIP and dual-luciferase reporter assays | ↓ | 48 |
| SK-N-SH cell line | Aβ_25-35_ | MiR-613 | Down-regulated | HDAC6 | Dual-luciferase reporter assay | ↓ | 49 |
| SH-SY5Y cell line | Aβ_42_ | MiR-19b-3p | Down-regulated | BACE1 | RIP and dual-luciferase reporter assays | ↓ | 41 |
| SH-SY5Y cell line | Aβ_42_ | MiR-106b-5p | Down-regulated | TXNIP | Dual-luciferase reporter assay | ↓ | 50 |
| PC12 cell line | Aβ_42_ | MiR-211 | Up-regulated | Ngn2 | Dual-luciferase reporter assay | ↑ | 51 |
| SH-SY5Y cell line | Aβ_42_ | MiR-143-3p | Up-regulated | NRG1 | Dual-luciferase reporter assay | ↑ | 52 |
| N2a cell line | APPswe mutant | MiR-455-3p | Up-regulated | APP | Dual-luciferase reporter assay | ↓ | 53 |
| SH-SY5Y cell line and PC12 cell line | Aβ_42_ | MiR-151-3p |  | DAPK-1 | Dual-luciferase reporter assay | ↓ | 38 |
| SH-SY5Y cell line and PC12 cell line | Aβ_42_ | MiR-151-3p |  | TP53 | Dual-luciferase reporter assay | ↓ | 38 |
| SK-N-SH cell line, SH-SY5Y cell line, and HEK293 cell line | Aβ_42_ | MiR-98-5p |  | SNX6 | Dual-luciferase reporter assay | ↑ | 54 |
| PC12 cell line and primary rat hippocampal neurons | Aβ_42_ | MiR-124 | Down-regulated |  |  | ↓ | 55 |
| PC12 cell line | Aβ_42_ | MiR-146a | Unchanged |  |  | ↑ | 43 |
| Primary rat cortical neurons | Aβ_42_ | MiR-146a | Up-regulated |  |  | ↑ | 43 |
| Primary mouse cortical neurons | Aβ | MiR-125b | Down-regulated |  |  | ↓ | 56 |
| SH-SY5Y cell line | Aluminum-maltolate | MiR-322 | Down-regulated |  |  | ↓ | 57 |
| SH-SY5Y cell line | Aβ_25-35_ | MiR-33 | Up-regulated |  |  | ↑ | 58 |
| SH‐SY5Y cell line | Aβ_42_ | MiR-21 | Up-regulated |  |  | ↓ | 59 |
| Human BMEC cell line | Aβ | Let-7g-5p | Down-regulated |  |  | ↓ | 60 |

Cellular AD models mainly constructed through the toxicity of Aβ in multiple nerve cells. Almost all the cell apoptosis related miRNAs in the cellular AD models abnormally expressed. Dual-luciferase reporter assay was the mainly method for the ‘miRNA-target’ identification. ‘↓’ presented cell apoptosis inhibition and ‘↑’ presented cell apoptosis promotion. Abbreviation: **BID**, BH3-interacting domain death agonist; **Lrp2**, Low-density lipoprotein receptor-related protein 2; **GSK-3β**, Glycogen synthase kinase 3 beta; **TNFAIP1**, BTB/POZ domain-containing adapter for CUL3-mediated RhoA degradation protein 2; **APP**, Amyloid-beta precursor protein; **BACE1**, Beta-secretase 1; **CDH13**, Cadherin-13; **Nrn1**, Neuritin; **DEK**, Protein DEK; **NOS1**, Nitric oxide synthase 1; **HDAC6**, Histone deacetylase 6; **TXNIP**, Thioredoxin-interacting protein; **Ngn2**, Neurogenin-2; **DAPK-1**, Death-associated protein kinase 1; **TP53**, Tumor protein p53; **SNX6**, Sorting nexin-6; **NRG1**, Pro-neuregulin-1, membrane-bound isoform.
