# Supplementary material for: NcRNAs: A synergistically antiapoptosis therapeutic tool in Alzheimer's disease
Source: CNS Neurosci Ther. 2023 Sep 22;30(4):e14476. doi: 10.1111/cns.14476 (PMC11017435; doi:10.1111/cns.14476)
Supplement: Supplementary file 2 — Table S2 [file CNS-30-e14476-s003.doc]

**Supplementary Table 2** Differentially expressed miRNAs in different AD animal models and their apoptosis regulation.

| AD animal model | Cellular AD model | | MiRNA | Expression | Target | Target validation methods | Apoptosis | References |
| --- | --- | --- | --- | --- | --- | --- | --- | --- |
|  | Cell type | Stimulation |  |  |  |  |  |  |
| APPswe/PSΔE9 mice | SH-SY5Y cell line and HEK 293T cell line |  | MiR-34a | Up-regulated | BCL-2 | Dual-luciferase reporter assay | ↑ | 87 |
| APP/PS1 mice | SH-SY5Y cell line and primary rat cortical neurons | Aβ_42_ | MiR-34a | Down-regulated | Cyclin D1 | Dual-luciferase reporter assay | ↓ | 88 |
| PS-2 mutant (N141I) mice | PC12 cell line and HEK 293T cell line | PS-2 (N141I) mutant | MiR-34a | Up-regulated | BCL-2 | Dual-luciferase reporter assay | ↑ | 89 |
| PS-2 mutant (N141I) mice | PC12 cell line and HEK 293T cell line | PS-2 (N141I) mutant | MiR-34a | Up-regulated | SIRT1 | Dual-luciferase reporter assay | ↑ | 89 |
| Micro-injection of 6-OHDA in mice | SH-SY5Y cell line and PC12 cell line | 6-OHDA | MiR-107 | Down-regulated | PDCD10 | Dual-luciferase reporter assay | ↓ | 84 |
| APPswe/PSΔE9 mice | Primary mouse cortical neurons | Aβ_25-35_ | MiR-429 | Up-regulated | SOX2 | Dual-luciferase reporter assay | ↑ | 80 |
| APPswe/PSΔE9 mice | Primary mouse cortical neurons | Aβ_25-35_ | MiR-429 | Up-regulated | BCL-2 | Dual-luciferase reporter assay | ↑ | 80 |
| Micro-injection of Aβ_25-35_ in rats | HEK 293T cell line |  | MiR-132-3p | Down-regulated | MAPK1 | Dual-luciferase reporter assay | ↓ | 85 |
| Micro-injection of Hcy in rats | SH-SY5Y cell line | Aβ_42_ | MiR-132-3p | Down-regulated | HNRNPU | Dual-luciferase reporter assays | ↓ | 86 |
| SAMP8 mice and micro-injection of Aβ_25-35_ in rats | Primary rat cortical neurons | Aβ_25-35_ | MiR-27a | Up-regulated | SOX8 | Dual-luciferase reporter assay | ↑ | 90 |
| APPswe/PS1dE9 mice | Primary mouse hippocampal neurons | Aβ_42_ | MiR-142-5p | Up-regulated | BAI3 | Dual-luciferase reporter assay | ↑ | 81 |
| Micro-injection of Aβ_42_ in rats | HEK 293T cell line |  | MiR-142-5p | Up-regulated | PTPN1 | Dual-luciferase reporter assay | ↑ | 81 |
| Micro-injection of Aβ_42_ in mice | Primary mouse hippocampal neurons |  | MiR-25 | Up-regulated | KLF2 | Dual-luciferase reporter assay | ↑ | 91 |
| APPswe/PSΔE9 mice | PC12 cell line | Aβ_25-35_ | MiR-200a-3p | Up-regulated | SIRT1 | Dual-luciferase reporter assay | ↑ | 92 |
| SAMP8 mice | PC12 cell line and Primary mouse hippocampal neurons | Aβ_42_ | MiR-873-5p | Down-regulated | HMOX1 | Dual-luciferase reporter assay | ↓ | 93 |
| Micro-injection of Aβ_25-35_ in rats | HEK 293T cell line |  | MiR-196a | Down-regulated | LRIG3 | Dual-luciferase reporter assay | ↓ | 94 |
| Micro-injection with scopolamine in mice | Primary mouse hippocampal neurons |  | MiR-98 | Down-regulated | HEY2 | Dual-luciferase reporter assay | ↓ | 95 |
| APP/PS1 mice | Primary mouse brain pericytes and HEK 293T cell line | Aβ_40_ | MiR-181a | Down-regulated | FOXO1 | Dual-luciferase reporter assay | ↓ | 96 |
| 5xFAD mice | Primary mouse cortical neurons and SH-SY5Y cell line | Aβ_42_ | MiR-16-5p | Up-regulated | BCL-2 | Dual-luciferase reporter assay | ↑ | 97 |
| APP/PS1 mice | Primary mouse hippocampal neurons | Aβ_40_ | MiR-338-5p | Down-regulated | BCL2L11 | Dual-luciferase reporter assay | ↓ | 98 |
| SAMP8 mice | SH-SY5Y cell line | APP Swedish mutant | MiR-340 | Down-regulated | BACE1 | Dual-luciferase reporter assay | ↓ | 99 |
| Micro-injection of streptozotocin in mice | PC12 cell line | Aβ | MiR-29c-3p | Down-regulated | BACE1 | Dual-luciferase reporter assay | ↓ | 100 |
| Micro-injection of Aβ_42_ in rats | Primary rat hippocampal neurons |  | MiR-10b-5p | Up-regulated | HOXD10 | Dual-luciferase reporter assay | ↑ | 101 |
| APPswe/PS1dE9 mice | SH-SY5Y cell line | Aβ_42_ | MiR-130a-3p | Down-regulated | DAPK-1 | Dual-luciferase reporter assay | ↓ | 102 |
| APP/PS1 mice | HEK293 cell line |  | MiR-216a-5p | Down-regulated | HMGB1 | Dual-luciferase reporter assay | ↓ | 103 |
| APPswe/PSΔE9 mice | PC12 cell line | Aβ_25-35_ | MiR-20b-5p | Up-regulated | RhoC | Dual-luciferase reporter assay | ↑ | 104 |
| APP/PS1 mice | Primary mouse brain pericytes | Aβ_40_ | MiR-485-5p | Down-regulated | PACS1 | Dual-luciferase reporter assay | ↓ | 105 |
| APPswe/PS1dE9 mice | HEK293 cell line |  | MiR-9-5p | Down-regulated | TPX2 | Dual-luciferase reporter assay | ↓ | 106 |
| APP/PS1 mice | Primary mouse microglial | Aβ_42_ | MiR-191-5p | Down-regulated | Map3k12 | Dual-luciferase reporter assay | ↓ | 107 |
| Micro-injection of Hcy in rats | PC12 cell line | Aβ_40_ | MiR-483-3p | Down-regulated | XPO1 | Dual-luciferase reporter assay | ↓ | 108 |
| 5×FAD mice | SH-SY5Y cell line and HEK 293T cell line | H_2_O_2_ | MiR-223 | Down-regulated | FOXO3 | Dual-luciferase reporter assay | ↓ | 109 |
| Tg2576 mice | SH-SY5Y cell line | Aβ_42_ | MiR-1273g-3p |  | mTOR | Dual-luciferase reporter assay | ↓ | 110 |
| AD mice | Primary hippocampal mouse neuronal stem cells | Aβ_42_ | MiR-668-3p | Up-regulated | OXR1 | Dual-luciferase reporter assay | ↑ | 111 |
| APPswe/PS1dE9 mice | Mouse hippocampal neurons |  | MiR-214-5p | Down-regulated | SUZ12 | RNA pull-down and dual-luciferase reporter assay | ↓ | 112 |
| APPswe/PS1dE9 mice | HEK 293T cell line |  | MiR-326 | Unchanged | VAV1 | Dual-luciferase reporter assay | ↓ | 113 |
| APPswe/PS1/E9 mice | U373MG cell line, SH-SY5Y cell line, primary  mouse astrocytes, and primary  mouse cortical neurons |  | MiR-135a | Up-regulated | THBS1 | Dual-luciferase reporter assay | ↑ | 114 |
| APPswe/PSΔE9 mice | N2a cell line | Aβ_42_ | MiR-22-3p | Down-regulated | MAPK14 | Dual-luciferase reporter assay | ↓ | 115 |
| APP/PS1 mice | HT22 cell line | Aβ_42_ | MiR-22-3p |  | SOX9 | Dual-luciferase reporter assay | ↓ | 116 |
| APP/PS1 mice | Primary mouse hippocampal neurons | Aβ_42_ | MiR-132-3p | Down-regulated |  |  | ↓ | 117 |
| Micro-injection of Aβ_42_ in rats |  |  | MiR-22 | Down-regulated |  |  | ↓ | 118 |
| Micro-injection of Al-malt in rats | SH-SY5Y cell line | Al-malt | MiR-19a | Down-regulated |  |  | ↓ | 119 |
| Micro-injection of Al-malt in rats | SH-SY5Y cell line | Al-malt | MiR-19b | Down-regulated |  |  | ↓ | 119 |
| APP/PS1 mice | Primary mouse cortical neurons, SH-SY5Y cell line, and HEK293 cell line | APPswe-GFP-transduced | MiR-107 | Down-regulated |  |  | ↓ | 83 |
| APP/PS1 mice | PC12 cell line | Aβ_25-35_ | MiR-22 | Up-regulated |  |  | ↓ | 120 |

AD animal models constructed by multiple methods. Cellular AD models mainly constructed by the toxicity of Aβ in multiple nerve cells. Almost all the cell apoptosis related miRNAs in AD animal models and/or cellular AD models abnormally expressed. Dual-luciferase reporter assay was the mainly method for the ‘miRNA-target’ identification. ‘↓’ presented cell apoptosis inhibition and ‘↑’ presented cell apoptosis promotion. Abbreviation: **BCL-2**, Apoptosis regulator BCL-2; **Cyclin D1**, G1/S-specific cyclin-D1; **SIRT1**, NAD-dependent protein deacetylase sirtuin-1; **PDCD10**, Programmed cell death protein 10; **SOX2**, Transcription factor SOX-2; **MAPK1**, Mitogen-activated protein kinase 1; **HNRNPU**, Heterogeneous nuclear ribonucleoprotein U; **SOX8**, Transcription factor SOX-8; **BAI3**, Adhesion G protein-coupled receptor B3; **PTPN1**, Tyrosine-protein phosphatase non-receptor type 1; **KLF2**, Krueppel-like factor 2; **HMOX1**, Heme oxygenase 1; **LRIG3**, Leucine-rich repeats and immunoglobulin-like domains protein 3; **HEY2**, Hairy/enhancer-of-split related with YRPW motif protein 2; **FOXO1**, Forkhead box protein O1; **BCL2L11**, Bcl-2-like protein 11; **BACE1**, Beta-secretase 1; **HOXD10**, Homeobox protein Hox-D10; **DAPK-1**, Death-associated protein kinase 1; **HMGB1**, High mobility group protein B1; **RhoC**, Rho-related GTP-binding protein RhoC; **PACS1**, Phosphofurin acidic cluster sorting protein 1; **TPX2**, Targeting protein for Xklp2; **Map3k12**, MAP3K12-binding inhibitory protein 1; **XPO1**, Exportin-1; **FOXO3**, Forkhead box protein O3; **mTOR**, Serine/threonine-protein kinase mTOR; **OXR1**, Oxidation resistance protein 1; **SUZ12**, Polycomb protein SUZ12; **VAV1**, Proto-oncogene vav; **THBS1**, Thrombospondin-1; **SOX9**, Transcription factor SOX-9; **MAPK14**, MAP kinase-activated protein kinase 14.
