# Supplementary material for: NcRNAs: A synergistically antiapoptosis therapeutic tool in Alzheimer's disease
Source: CNS Neurosci Ther. 2023 Sep 22;30(4):e14476. doi: 10.1111/cns.14476 (PMC11017435; doi:10.1111/cns.14476)
Supplement: Supplementary file 3 — Table S3 [file CNS-30-e14476-s002.doc]

**Supplementary Table 3** Differentially expressed miRNAs in AD patients and their apoptosis regulation.

| Materials from AD patients | AD animal model | Cellular AD model | | MiRNA | Expression | Target | Target validation methods | Apoptosis | References |
| --- | --- | --- | --- | --- | --- | --- | --- | --- | --- |
|  |  | Cell type | Stimulation |  |  |  |  |  |  |
| Temporal cortical areas and CA1 hippocampal neurons | APP/PS1 mice | Primary mouse/rat cerebrocortical neurons and PC12 cell line |  | MiR-132 | Down-regulated | PTEN | Dual-luciferase reporter assay | ↓ | 132 |
| Temporal cortical areas and CA1 hippocampal neurons | APP/PS1 mice | Temporal cortical areas and CA1 hippocampal neurons of human AD brains |  | MiR-132 | Down-regulated | FOXO3a | Dual-luciferase reporter assay | ↓ | 132 |
| Frozen human postmortem brain specimens |  | Primary human cortical neurons |  | MiR-132 | Up-regulated | GTDC-1 | Dual-luciferase reporter assay | ↑ | 139 |
| Temporal cortical areas and CA1 hippocampal neurons | APP/PS1 mice | Primary mouse/rat cerebrocortical neurons and PC12 cell line |  | MiR-212 | Down-regulated | PTEN | Dual-luciferase reporter assay | ↓ | 132 |
| Temporal cortical areas and CA1 hippocampal neurons | APP/PS1 mice | Primary mouse/rat cerebrocortical neurons and PC12 cell line |  | MiR-212 | Down-regulated | FOXO3a | Dual-luciferase reporter assay | ↓ | 132 |
| Brain  specimens | APP/PS1 mice | HCN-2 cell line |  | MiR-124-3p | Down-regulated | CAPN1 | Dual-luciferase reporter assay | ↓ | 142 |
| Blood plasma | APP/PS1 and SAMP8 mice | SH-SY5Y cell line | APPswe mutant | MiR-200a-3p | Down-regulated | PRKACB | Dual-luciferase reporter assay | ↓ | 143 |
| Blood plasma | APP/PS1 and SAMP8 mice | SH-SY5Y cell line | APPswe mutant | MiR-200a-3p | Down-regulated | BACE1 | Dual-luciferase reporter assay | ↓ | 143 |
| CSF | APP/PS1 mice | SH-SY5Y cell line |  | MiR-539-5p | Down-regulated | APP | Dual-luciferase reporter assay | ↓ | 135 |
| CSF | APP/PS1 mice | SH-SY5Y cell line |  | MiR-539-5p | Down-regulated | CAV1 | Dual-luciferase reporter assay | ↓ | 135 |
| CSF | APP/PS1 mice | SH-SY5Y cell line |  | MiR-539-5p | Down-regulated | GSK-3β | Dual-luciferase reporter assay | ↓ | 135 |
| Serum samples |  | Primary mouse cortical neurons and N2a cell line | Aβ_25-35_ | MiR-125b-5p | Down-regulated | BACE1 | Dual-luciferase reporter assay | ↓ | 141 |
| Serum samples |  | Primary mouse cortical neurons and N2a cell line | Aβ_25-35_ | MiR-34a-5p | Down-regulated | BACE1 | Dual-luciferase reporter assay | ↓ | 141 |
| Plasma |  | SH-SY5Y cell line and IMR-32 cell line | Aβ_25-35_ | MiR-212 | Down-regulated | PDCD4 | Dual-luciferase reporter and RNA pull-down assays | ↓ | 133 |
| CSF |  | SH-SY5Y cell line | Aβ_25-35_ | MiR-107 | Down-regulated | FGF7 | Dual-luciferase reporter assay | ↓ | 144 |
| Plasma | APP/PS1 and 5×FAD mice | SH-SY5Y cell line and HEK293 cell line | APPswe mutant | MiR-30a-5p | Up-regulated | ADAM10 | Dual-luciferase reporter assay | ↑ | 145 |
| Plasma | APP/PS1 and 5×FAD mice | SH-SY5Y cell line and HEK293 cell line | APPswe mutant | MiR-30a-5p | Up-regulated | SIRT1 | Dual-luciferase reporter assay | ↑ | 145 |
| Human postmortem brains |  | Primary human and rat cortical neurons |  | MiR-26b | Up-regulated | Rb1 | Dual-luciferase reporter assay | ↑ | 136 |
| CSF | APPswe/PS1dE9 mice | Primary mouse hippocampal neurons, N2a cell line and SH-SY5Y cell line |  | MiR-299-5p | Down-regulated | Atg5 | Dual-luciferase reporter assay | ↓ | 146 |
| Frontal brain cortices |  | PC12 cell line | Aβ_42_ | MiR-16 | Down-regulated | BACE1 | Dual-luciferase reporter assay | ↓ | 147 |
| Serum |  | SH-SY5Y cell line | Aβ_25-35_ | MiR-133b | Down-regulated | EGFR | Dual-luciferase reporter assay | ↓ | 148 |
| Human postmortem brain |  | HEK293 cell line and N2a cell line | Human tau and APP695 | MiR-425-5p | Up-regulated | HSPB8 | Dual-luciferase reporter assay | ↑ | 137 |
| Serum |  | PC12 cell line and SH-SY5Y cell line | Aβ_25-35_ | MiR-193a-3p | Down-regulated | PTEN | Dual-luciferase reporter assay | ↓ | 149 |
| Brains | APP/PS1 mice | SH-SY5Y cell line | APPswe mutant | MiR-335-5p | Down-regulated | JNK3 | Dual-luciferase reporter assay | ↓ | 150 |
| Serum | Micro-injection of Aβ_42_ in mice | Primary mouse hippocampal neurons | Aβ_42_ | MiR-129 | Down-regulated | YAP1 | Dual-luciferase reporter assay | ↓ | 134 |
| Serum |  | PC12 cell line and 293 cell line | Aβ | MiR-202 | Down-regulated | APP | Dual-luciferase reporter assay | ↓ | 151 |
| Serum |  | SH-SY5Y cell line | Aβ_25-35_ | MiR-148a-3p | Down-regulated | ROCK1 | Dual-luciferase reporter assay | ↓ | 152 |
| Serum |  | SH-SY5Y cell line | Aβ_25-35_ | MiR-24-3p | Up-regulated | KLF8 | Dual-luciferase reporter assay | ↑ | 153 |
| Brain frontal cortex, precentral gyrus, CSF, and plasma | 5×FAD mice | Primary mouse glial cells, primary mouse cortical neurons, and HEK 293T cell line | Aβ_42_ | MiR-485-3p | Up-regulated | CD36 | Dual-luciferase reporter assay | ↑ | 138 |
| Serum | APP/PS1 mice | BV-2 cell line and HT22 cell line | Aβ | MiR-590-5p | Down-regulated | PELI1 | Dual-luciferase reporter assay | ↓ | 154 |
| Serum |  | SH-SY5Y cell line | Aβ_25-35_ | MiR-381-3p | Down-regulated | PTGS2 | Dual-luciferase reporter assay | ↓ | 155 |
| Plasma | APP/ PS1 and SAMP8 mice | SH-SY5Y cell line and HEK293 cell line | APPswe mutant | MiR-23b-3p | Down-regulated | GSK-3β | Dual-luciferase reporter assay | ↓ | 156 |
| CSF | SAMP8 mice | Primary mouse hippocampal neurons and SH-SY5Y cell line |  | MiR-214-3p | Down-regulated | Atg12 | Dual-luciferase reporter assay | ↓ | 157 |
| Serum |  | SH-SY5Y cell line | Aβ_40_ | MiR-223 | Down-regulated |  |  | ↓ | 158 |
| CSF |  | N2a cell line | APPSwe/Δ9 mutant | MiR-125b-5p | Up-regulated |  |  | ↑ | 140 |
| CSF |  | Primary mouse cortical neurons and SH-SY5Y cell line |  | Let-7b | Up-regulated |  |  | ↑ | 159 |
| CSF |  | Primary mouse cortical neurons and SH-SY5Y cell line |  | Let-7g | Unchanged |  |  | ↑ | 159 |
| CSF |  | Primary mouse cortical neurons and SH-SY5Y cell line |  | Let-7f |  |  |  | ↑ | 159 |
| CSF |  | SK-N-SH cell line | Aβ_40_ | Let-7b | Up-regulated |  |  | ↑ | 160 |

AD animal models constructed by multiple methods. Cellular AD models mainly constructed by the toxicity of Aβ in multiple nerve cells. Almost all the cell apoptosis related miRNAs in AD patients and/or AD animal models and/or cellular AD models abnormally expressed. Dual-luciferase reporter assay was the mainly method for the ‘miRNA-target’ identification. ‘↓’ presented cell apoptosis inhibition and ‘↑’ presented cell apoptosis promotion. Abbreviation: **CSF**, Cerebrospinal fluid; **PTEN**, Phosphatidylinositol 3,4,5-trisphosphate 3-phosphatase and dual-specificity protein phosphatase PTEN; **FOXO3a**, Forkhead box O3A; **GTDC-1**, Glycosyltransferase-like domain-containing protein 1; **CAPN1**, Calpain-1 catalytic subunit; **PRKACB**, cAMP-dependent protein kinase catalytic subunit beta; **BACE1**, Beta-secretase 1; **APP**, Amyloid-beta precursor protein; **CAV1**, Caveolin 1； **GSK-3β**, Glycogen synthase kinase 3 beta; **PDCD4**, Programmed cell death protein 4; **FGF7**, Fibroblast growth factor 7; **ADAM10**, Disintegrin and metalloproteinase domain-containing protein 10; **SIRT1**, NAD-dependent protein deacetylase sirtuin-1; **Rb1**, Retinoblastoma-associated protein; **Atg5**, Autophagy protein 5; **EGFR**, Epidermal growth factor receptor; **HSPB8**, Heat shock protein beta-8; **JNK3**, Mitogen-activated protein kinase 10; **YAP1**, Yes1 associated transcriptional regulator; **ROCK1**, Rho associated coiled-coil containing protein kinase 1; **KLF8**, Krueppel-like factor 8; **CD36**, Platelet glycoprotein 4; **PELI1**, E3 ubiquitin-protein ligase pellino homolog 1; **PTGS2**, Prostaglandin G/H synthase 2; **Atg12,** Autophagy protein 12.
