# Supplementary material for: NcRNAs: A synergistically antiapoptosis therapeutic tool in Alzheimer's disease
Source: CNS Neurosci Ther. 2023 Sep 22;30(4):e14476. doi: 10.1111/cns.14476 (PMC11017435; doi:10.1111/cns.14476)
Supplement: Supplementary file 4 — Table S4 [file CNS-30-e14476-s004.doc]

**Supplementary Table 4** Basing on mRNA as the core, other ncRNAs act synergistically in anti-apoptosis in AD.

| LncRNA | CircRNA | MiRNA | **MRNA** | Apoptosis |
| --- | --- | --- | --- | --- |
| ATB**↓** |  | miR-200**↑** | **ZNF217** | Inhibition |
| SNHG1**↓** |  | miR-316-3p**↑** |
|  | LPAR1**↓** | miR-212-3p**↑** |
|  |  | miR-9-5p**↑** | **GSK-3β** | Inhibition |
|  |  | miR-23b-3p**↑** |
|  |  | miR-539-5p**↑** |
|  |  | miR-34a**↓** | **SIRT1** | Inhibition |
|  |  | miR-30a-5p**↓** |
|  |  | miR-200a-3p**↓** |
|  |  | miR-29c-3p**↑** | **TNFAIP1** | Inhibition |
|  | 0002945**↓** | miR-431-5p**↑** |
|  |  | miR-137**↑** |
|  |  | miR-455-3p**↑** | **APP** | Inhibition |
|  |  | miR-539-5p**↑** |
|  |  | miR-202**↑** |
|  |  | miR-16-5p |
|  | 0002594**↓** | miR-139-5p**↑** |

‘**↓**’ represents down-regulating the expression of the ncRNA and ‘**↑**’ represents up-regulating the expression of the ncRNA. Simultaneously controlling the expression of different ncRNAs plays a synergistic anti-apoptosis function in AD, basing on mRNA as the core. Abbreviations: **ZNF217**, Zinc finger protein 217; **GSK-3β**, Glycogen synthase kinase 3 beta; **SIRT1**, NAD-dependent protein deacetylase sirtuin-1; **TNFAIP1**, BTB/POZ domain-containing adapter for CUL3-mediated RhoA degradation protein 2; **APP**, Amyloid-beta precursor protein.
