# Supplementary material for: NcRNAs: A synergistically antiapoptosis therapeutic tool in Alzheimer's disease
Source: CNS Neurosci Ther. 2023 Sep 22;30(4):e14476. doi: 10.1111/cns.14476 (PMC11017435; doi:10.1111/cns.14476)
Supplement: Supplementary file 5 — Table S5 [file CNS-30-e14476-s005.doc]

**Supplementary Table 5** Basing on miRNA as the core, other ncRNAs act synergistically in anti-apoptosis in AD.

| LncRNA | CircRNA | **MiRNA** | MRNA | Apoptosis |
| --- | --- | --- | --- | --- |
| SOX21-AS1**↓** |  | **miR-137↑** | MAPK1 | Inhibition |
| BACE1-AS**↓** |  |  | HNRNPU |  |
|  |  |  | PTEN |  |
|  |  |  | FOXO3a |  |
|  |  |  | GTDC-1 |  |

‘**↓**’ represents down-regulating the expression of the ncRNA and ‘**↑**’ represents up-regulating the expression of the ncRNA. Simultaneously controlling the expression of different ncRNAs plays a synergistic anti-apoptosis function, basing on miRNA as the core. Abbreviations: **MAPK1**, Mitogen-activated protein kinase 1; **HNRNPU**, Heterogeneous nuclear ribonucleoprotein U; **PTEN**, Phosphatidylinositol 3,4,5-trisphosphate 3-phosphatase and dual-specificity protein phosphatase PTEN; **FOXO3a**, Forkhead box protein O3A; **GTDC-1**, Glycosyltransferase-like domain-containing protein 1.
